# Experimental Identification of the Roles of Fe, Ni and Attapulgite in Nitroreduction and Dechlorination of p-Chloronitrobenzene by Attapulgite-Supported Fe/Ni Nanoparticles

**DOI:** 10.3390/ma15031254

**Published:** 2022-02-08

**Authors:** Jing Liang, Junwen Wang, Hong Liu, Emmanuella Anang, Xianyuan Fan

**Affiliations:** 1 College of Environmental Engineering, Nanjing Polytechnic Institute, Nanjing 210048, China; liangjing@njpi.edu.cn; 2 College of Resource and Environmental Engineering, Wuhan University of Science and Technology, Wuhan 430081, China; wangjunwen@wust.edu.cn (J.W.); anangemmanuella@gmail.com (E.A.); fanxianyuan@wust.edu.cn (X.F.); 3 Hubei Key Laboratory for Efficient Utilization and Agglomeration of Metallurgic Mineral Resources, Wuhan University of Science and Technology, Wuhan 430081, China

**Keywords:** attapulgite-supported nFe/Ni nanoparticles, attapulgite, Fe, Ni, p-chloronitrobenzene

## Abstract

The porous-material loading and noble-metal doping of nanoscale zero-valent iron (nFe) have been widely used as countermeasures to overcome its limitations. However, few studies focused on the experimental identification of the roles of Fe, the carrier and the doped metal in the application of nFe. In this study, the nitroreduction and dechlorination of p-chloronitrobenzene (p-CNB) by attapulgite-supported Fe/Ni nanoparticles (ATP-nFe/Ni) were investigated and the roles of Fe, Ni and attapulgite were examined. The contributions of Ni are alleviating the oxidization of Fe, acting as a catalyst to trigger the conversion of H_2_ to H*(active hydrogen atom) and promoting electron transfer of Fe. The action mechanisms of Fe in reduction of -NO_2_ to -NH_2_ were confirmed to be electron transfer and to produce H_2_ via corrosion. When H_2_ is catalyzed to H* by Ni, the production H* leads to the nitroreduction. In additon, H* is also responsible for the dechlorination of p-CNB and its nitro-reduced product, p-chloroaniline. Another corrosion product of Fe, Fe^2+^, is incapable of acting in the nitroreduction and dechlorination of p-CNB. The roles of attapulgite includes providing an anoxic environment for nFe, decreasing nFe agglomeration and increasing reaction sites. The results indicate that the roles of Fe, Ni and attapulgite in nitroreduction and dechlorination of p-CNB by ATP-nFe/Ni are crucial to the application of iron-based technology.

## 1. Introduction

Chloronitrobenzene, a kind of chloro-organic compound with high toxicity and resistance to microbial degradation, is widely used as an intermediate in the chemical syntheses of pesticides, dyes, antioxidants and herbicides [1,2,3,4]. The chloronitrobenzenes include three isomers, among which, p-chloronitrobenzene (p-CNB) is more readily present in the environment because it is slightly soluble in water [5,6]. The improper disposal and accidental spills of p-CNB have led to the prevalent soil and water contamination. Hence, several attempts, such as catalytic reduction, electrolysis and ozonation techniques have been performed by previous studies [7,8,9].

Zero-valent iron technology, the premier cost-effective technology adopted in the remediation of contaminated water and soil, has been advanced over the years [10,11]. It has taken the form of nanoscale zero-valent iron (nFe) in order to increase reactivity as compared to the micron-sized and granular zero-valent iron [11,12]. However, a major limitation of nFe application is evident in its rapid aggregation [13]. To address this issue, porous materials, such as bentonite, resin, carbon nanotubes, zeolite and activated carbon, have been explored as carriers to enhance the stability of nFe [14,15,16,17,18]. Additionally, to further improve the reactivity, a secondary metal, such as Ni and Pd, is employed to deposit on the surface of nFe, and the resulting bimetallic nanoparticles have been verified to be more effective in the dechlorination of various chloro-organics than the monometallic counterpart [19,20,21]. For instance, a 45% higher removal efficiency of pentachlorophenol was achieved by nFe/Ni bimetal, compared to nFe alone [22]. 

Even though some researchers have reported the reduction and dechlorination of many kinds of chloro-organics by nFe and bimetallic composites, much focus is placed on the dechlorination efficiency, pathway and kinetics [16,23,24,25,26]. The mechanism by which Fe acts in reduction and dechlorination is often explained using three theoretical possibilities: serving as an electron donor and producing H_2_ and Fe^2+^ via corrosion, both of which are able to act as reductants. There are also three interpretations of the role played by the second metal: (i) depositing on the surface of Fe nanoparticles, thus weakening the Fe oxidation; (ii) catalyzing H_2_ into an active hydrogen atom; (iii) acting as the cathode of the Fe-Ni galvanic cell, which promotes the electron transfer from Fe to chloro-organics. As for the role of the carrier, most studies attribute it to the steric hindrance of the porous material preventing the agglomeration of nFe. To our best knowledge, few studies have focused on the identification of the roles of Fe, the carrier and the doped metal in the reduction and dechlorination of chloro-organics through experimental studies.

In this study, we adopt p-CNB that has two functional groups of different redox chemistry (-NO_2_ and C-Cl) as the model pollutant of chloro-organics, and Ni as the second metal deposited on the nFe. Attapulgite (ATP), a kind of clay characterized by a nano-fiber rod with a diameter of 20–70 nm and a length of 0.5–5 μm, is employed as the carrier to synthesize attapulgite-supported Fe/Ni nanoparticles (ATP-nFe/Ni). The objectives of this work are to investigate: (i) the real action mechanisms of Fe for the nitroreduction (the -NO_2_ is reduced to -NH_2_) and dechlorination (the C-Cl bond is broken) of p-CNB, and the corresponding roles of Ni; (ii) contributions of attapulgite during the nitroreduction and dechlorination of p-CNB by ATP-nFe/Ni. In addition, the pathway and kinetics in the reaction are also examined.

## 2. Materials and Methods

### 2.1. Materials and Chemicals

Attapulgite powder was obtained from Anhui Mingmei Mineral Co., Ltd. (Mingguang, China). The chemicals used in the experiment were p-chloronitrobenzene, p-chloroaniline, aniline, nickel sulfate (NiSO_4_·6H_2_O), sulfuric acid (H_2_SO_4_), ferrous sulfate (FeSO_4_·7H_2_O) and sodium borohydride (NaBH_4_), all of which were analytical grade and purchased from Sinopharm Chemical Reagent Co., Ltd. (Shanghai, China) 

### 2.2. Preparation of Materials

The liquid phase reduction method was used to prepare ATP-nFe/Ni [23,27]. Specifically, 2.24 g of ATP and 20 mL FeSO_4_ solution (1 M) were added to a three-necked flask and the resulting mixture was mechanically stirred (150 rpm) for 30 min in a nitrogen (N_2_) atmosphere. Subsequently, 80 mL of a NaBH_4_ solution (0.5 M) was dropwise to the mixture (about 100 drops per minute). After sufficient reaction, 0.8 mL of a NiSO_4_ solution (0.5 M) was added to the mixture and stirred continuously for 20 min. The prepared material was vacuum-filtered and washed with deionized water for 2–3 times, and dried in a freeze dryer for 16 h. The mass ratio of ATP:Fe:Ni in the resulting composite was 2:1:0.02. The same procedure was used to obtain Fe/Ni nanoparticles (nFe/Ni), ATP-supported Fe nanoparticles (ATP-nFe) and ATP-supported Ni nanoparticles (ATP-nNi), except that the ATP, NiSO_4_ solution and FeSO_4_ solution was not added, respectively. The unsupported nFe was prepared by reacting the 80 mL NaBH_4_ solution (0.5 M) (dropwise) with the 20 mL FeSO_4_ solution (1 M) in a nitrogen atmosphere.

### 2.3. Batch Experiments

Batch experiments for the nitroreduction and dechlorination of p-CNB were conducted in a stoppered conical flask of 500 mL. Prior to each experiment, the pH of solution was adjusted by using 0.1 M H_2_SO_4_ or 1.0 M NaOH. To each conical flask containing 200 mL solution of p-CNB with a concentration of around 10 mg/L, 0.1 g of freshly prepared ATP-nFe/Ni (or other material) was added. The reactors were shaken (250 rpm) continuously at 25 °C for 2 h. Each experiment was carried out in triplicate. The concentration of p-CNB and its nitroreduction and dechlorination products, parachloroaniline (p-CAN) and aniline (AN), were analyzed by high-performance liquid chromatography (UltiMate 3000, Dionex, Waltham, MA, USA) fitted with a C-18 column. The mobile phase of methanol and water with a ratio of 70:30 (V:V) were delivered at a rate of 1.0 mL/min. The wavelength of the UV detector was set at 270 nm, 240 nm and 230 nm for the analysis of p-CNB, p-CAN and AN, respectively.

### 2.4. Material Characterizations

The morphologies of synthesized materials, such as ATP-nFe/Ni and nFe/Ni, were characterized using a transmission electron microscope (TEM) (Tecnai G2 F30 S-Twin, FEI, Hillsboro, OR, USA). The crystalline state was identified using an X-ray diffractometer (XRD) (D/MAX-2500, Rigaku Co., Tokyo, Japan) with Mo Kα irradiation at 45 kV and 250 mA. The Brunner Emmett Teller (BET) specific surface area, pore-volume and pore size distribution of the synthesized materials were determined by a specific surface area analyzer (ASAP 2020, Micromeritics Instrument Co., Ltd., Atlanta, GA, USA). The redox potential (ORP or Eh) of deionized water, FeSO_4_ solution, p-CNB solution and synthesized materials were measured using an electrochemical workstation (CHI-660E, CH Instruments, Inc., Austin, TX, USA). The individual Eh measurements lasted for 65 min, and their corresponding Eh values (as determined by the electrochemical workstation) were analyzed. The content of Fe in the material was measured by a device (FH-1, INESA Co., Shanghai, China), which is based on the reaction of Fe with dilute hydrochloric. By measuring the H_2_ generated by the reaction, the content of Fe can be determined. The concentration of dissolved H_2_ was measured using a portable dissolved hydrogen meter (ENH 1000, Trustlex, Okayama, Japan).

## 3. Results and Discussion

### 3.1. Characterizations of ATP, nFe/Ni and ATP-nFe/Ni 

**Figure 1:**
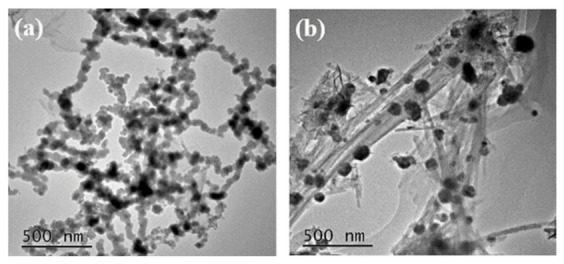
TEM images of nFe/Ni (**a**) and ATP-nFe/Ni (**b**).

The representative morphology of nFe/Ni and ATP-nFe/Ni are shown in Figure 1. The approximately spherical particles that are characteristic of the nFe are found to be part of a chain-like aggregation of nFe/Ni. The high surface energy and magnetic properties of nFe particles are responsible for the aggregation of nFe/Ni to form relatively larger clusters (Chen and Lee) [28]. The aggregated morphology of nFe/Ni particles become disintegrated with the introduction of ATP into the composite (Figure 1b). As can be observed in Figure 1b, the ATP, which is characterized by interwoven fibers, provides support for the nFe/Ni particles, whose aggregation was broken. Hence, a considerable proportion of the nFe/Ni particles with a diameter range of 30 to 75 nm, consistent with the reported diameter in works of literature (<100 nm), are dispersed on the ATP surface [29,30]. 

**Figure 2:**
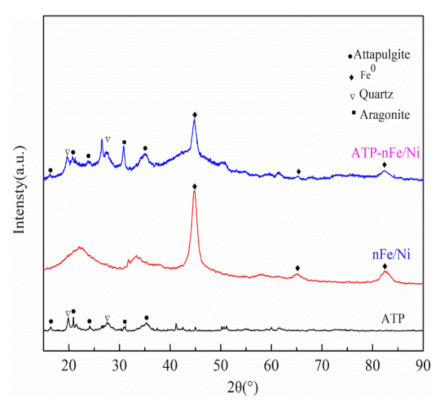
XRD patterns of ATP, nFe/Ni and ATP-nFe/Ni.

Figure 2 shows the XRD patterns of ATP, nFe/Ni and ATP-nFe/Ni. The diffraction peaks at 16.5°, 21.4°, 24.1° and 35.8° (JCPDS 20-0688) are characteristic of ATP. The ATP and ATP-nFe/Ni patterns also demonstrate the presence of impurities, such as quartz (20.9° and 26.7°; JCPDS 01-0649) and aragonite (31.0°; JCPDS 01-0628). Additionally, the XRD patterns of nFe/Ni and ATP-nFe/Ni depict the presence of Fe° (44.7°, 65.1° and 82.4°; JCPDS 06-0696). The diffraction peak of Ni was not observed due to the relatively low content of Ni in nFe/Ni and ATP-nFe/Ni.

The BET specific surface area and pore parameters of ATP, nFe/Ni and ATP-nFe/Ni were measured and presented in Table 1. The BET specific surface area of ATP (110.16 m^2^/g) is higher than that of nFe/Ni and ATP-nFe/Ni. Meanwhile, the higher pore volume (0.3160 cm^3^/g) and a relatively low pore diameter (11.47 nm) of ATP are recorded. Furthermore, the BET specific surface area of nFe/Ni is 14.15 m^2^/g, with a pore volume and pore diameter of 0.0920 cm^3^/g and 26.01 nm, respectively. However, the BET specific surface area of ATP-nFe/Ni is 101.33 m^2^/g, which is about 7.2 times larger than that of nFe/Ni. Moreover, the specific surface area of the mesoporous, S_meso_, which is beneficial to the diffusion of contaminants [31,32], increased from 13.39 m^2^/g to 77.69 m^2^/g. A similar tendency is also observed for the pore volume.

**Figure 3:**
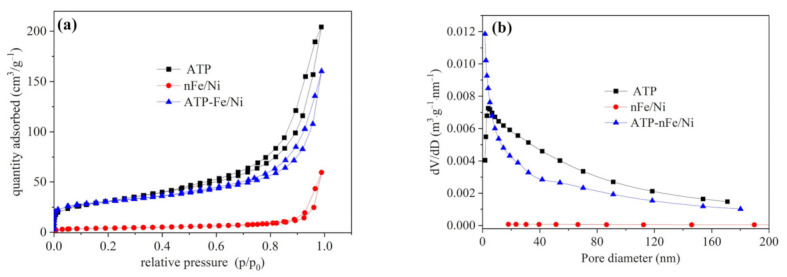
N_2_ adsorption–desorption isotherms (**a**) and pore diameter distribution curves (**b**) of ATP, nFe/Ni and ATP-nFe/Ni.

Figure 3 shows the N_2_ adsorption–desorption isotherms (a) and pore diameter distribution curves (b) of ATP, nFe/Ni and ATP-nFe/Ni. It can be found that the adsorption isotherms of the three materials all belong to type IV isotherms with hysteretic loops. However, the hysteresis of nFe/Ni occurs in the region of p/p_0_ close to 1.0, indicating that the pores in nFe/Ni are formed by particle accumulation. When nFe/Ni is loaded by ATP, the hysteretic loops of ATP-nFe/Ni are similar to that of ATP, suggesting that the adsorption characteristics and pore structure of ATP-nFe/Ni are more similar to ATP.

As can be observed from Figure 3b, the volume of macroporous (pore diameter > 50 nm), mesoporous (pore diameter = 2–50 nm) and micropore (pore diameter < 2 nm) in nFe/Ni are relatively small. However, the pore diameter distribution curve of ATP has a peak at 3.8 nm, indicating that the mesoporous with a diameter of 3.8 nm produces the largest pore volume compared with the macropores and micropores. The pore size distribution curve of ATP-nFe/Ni is different from that of both ATP and nFe/Ni. It shows that the pore volume of smaller pore size tends to increase, while the pore volume of mesoporous and macropore decreases compared with that of ATP. This result may be caused by the filling of mesoporous and macropore of ATP with Fe/Ni nanoparticles.

### 3.2. Removal, Nitroreduction and Dechlorination of p-CNB by Different Materials 

**Figure 4:**
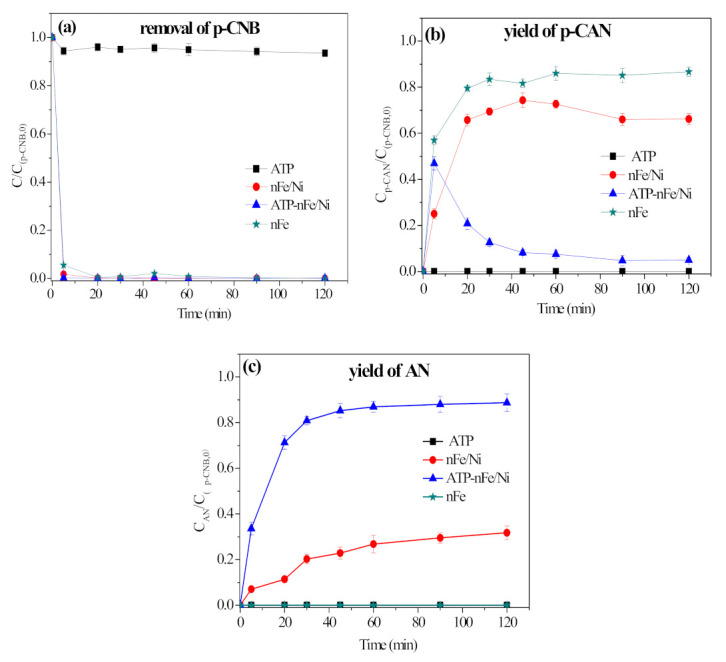
Removal of p-CNB (**a**), nitroreduction of p-CNB (yield of p-CAN) (**b**) and dechlorination of p-CNB (yield of AN) (**c**) by different materials (reaction conditions: concentration of p-CNB = 10 mg/L, dosage of material = 0.5 g/L and initial pH = 5.6 ± 0.1).

The removal, nitroreduction and dechlorination of p-CNB by ATP, nFe, nFe/Ni and ATP-nFe/Ni are presented in Figure 4. The removal rate of p-CNB by ATP was only about 6.5% (Figure 4a). In Figure 4b,c, no nitroreduction and dechlorination products (p-CAN and AN) were achieved by ATP, as a constant rate of 0% was reached up to 120 min, suggesting that p-CNB can only be adsorbed by ATP. The removal efficiency of p-CNB by nFe was nearly 100% at 5 min, and this persisted until 120 min (Figure 4a). Moreover, a fast transformation of p-CNB to p-CAN was realized by nFe (Figure 4b) and a rate of 86.7% was achieved at 120 min. However, the dechlorination product, AN, was not detectable (Figure 4c), hence, p-CNB and p-CAN cannot undergo dechlorination by nFe.

For nFe/Ni and ATP-nFe/Ni, both can achieve dechlorination and nitroreduction. However, the efficiency of nFe/Ni and ATP-nFe/Ni are different. The nitroreduction rates of p-CNB by nFe/Ni and ATP-nFe/Ni reached 66.2% and 5.0% at 120 min, respectively (Figure 4b), and the dechlorination rate of p-CNB (yield of AN) reached 31.2% and 88.8% at 120 min, respectively (Figure 4c). It is worth noting that the nitroreduction efficiency of ATP-nFe/Ni is lower than that of nFe/Ni, which was caused by the rapid dechlorination of p-CAN to AN by ATP-nFe/Ni.

### 3.3. Pathway and Kinetics of p-CNB Nitroreduction and Dechlorination by ATP-nFe/Ni

**Figure 5:**
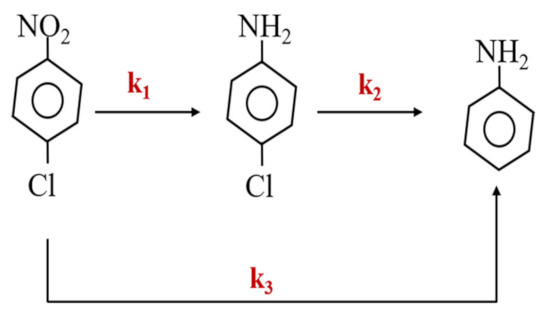
Pathway of p-CNB nitroreduction and dechlorination by ATP-nFe/Ni(k_1_, k_2_ and k_3_ denote the corresponding rate constants for the transformation of p-CNB, p-CAN and AN, respectively).

The probable pathways of p-CNB nitroreduction and dechlorination by ATP-nFe/Ni could be simplified to the sequence of steps as shown in Figure 5. The reaction rate of each substrate compound can be expressed as follows:

(1) $${\;C}_{p-\mathrm{CNB}}{=C}_{p-\mathrm{CNB},0}e^{{-(k}_{1}{+k}_{3})t}$$

(2) $${\;C}_{p-\mathrm{CAN}}{=C}_{p-\mathrm{CNB},0}\times \left[\frac{k_{1}}{k_{2}-\left(k_{1}+k_{3}\right)}\left(e^{{-(k}_{1}{+k}_{3})t}-e^{-k_{2}t}\right)\right]$$

(3) $$\;\;\;\;\;\;\;\;\;\;\;\;\;\;\;\;\;\;C_{\mathrm{AN}}{=C}_{p-\mathrm{CNB},0}{-C}_{p-\mathrm{CAN}}$$

where k_1_, k_2_ and k_3_ denote the corresponding rate constants for the transformation of p-CNB, p-CAN and AN, respectively. The calculation of k_1_, k_2_ and k_3_ was completed with the aid of the non-linear least square method [33]. The k values were found to be: k_1_ = 0.7853 min^−1^, k_2_ = 0.0159 min^−1^ and k_3_ = 0.5139 min^−1^. The rate constant for the simultaneous reduction of -NO_2_ and dechlorination of C-Cl bond (k_3_) is 0.5139 min^−1^, indicating that p-CNB can be dechlorinated directly. Moreover, k_1_ is much greater than k_2_, suggesting that the reduction of -NO_2_ is easier than the reductive dechlorination of the C-Cl bond, and the dechlorination from p-CAN to AN is the rate-limiting step of the p-CNB reduction by ATP-nFe/Ni [34]. Furthermore, the rate constant for the p-CNB dechlorination to AN is greater than that of p-CAN to AN (k_2_ = 0.0159 min^−1^, and k_3_ = 0.5139 min^−1^), which may be caused by the possible steric effect and the electron-withdrawing behavior of -Cl, -NH_2_ and -NO_2_.

In summary, the dominant pathway of p-CNB nitroreduction and dechlorination by ATP-nFe/Ni can be described as the nitroreduction of p-CNB to p-CAN, as well as the continuous Cl- elimination to form AN. The side route is the simultaneous nitroreduction and dechlorination of p-CNB with AN as the final product.

### 3.4. The Role of ATP in Nitroreduction and Dechlorination of p-CNB by ATP-nFe/Ni

As can be observed from Figure 4, the dechlorination efficiency of p-CNB by ATP-nFe/Ni is 57.6% higher than that by nFe/Ni, while the theoretical mass fraction of Fe in ATP-nFe/Ni and nFe/Ni is 33.3% and 98.0%, respectively. From the yield of p-CAN, the nitroreduction efficiency of nFe/Ni is higher than that in ATP-nFe/Ni; this can be attributed to the rapid dechlorination of p-CAN by ATP-nFe/Ni. The lower dosage of Fe/Ni nanoparticles in ATP-nFe/Ni can achieve a greater rate of nitroreduction and dechlorination than the higher dosage of unsupported Fe/Ni nanoparticles (the dosage of nFe/Ni in ATP-nFe/Ni and unsupported nFe/Ni is 0.17 g/L and 0.5 g/L, respectively), which should be attributed to the load of ATP. 

First of all, the clusters of Fe/Ni nanoparticles are well disaggregated due to ATP load, which has been confirmed by TEM images. Some nFe/Ni in ATP-Fe/Ni existed as an individual nanoparticle, which resulted from the adsorption and cation exchange of ATP towards Fe^2+^. The adsorbed and exchanged Fe^2+^ was transformed into Fe nanoparticles after its reduction by NaBH_4_, yet, the magnetic interaction among Fe nanoparticles was separated by ATP. Besides, the aggregations of the Fe nanoparticles formed at exchangeable sites were also limited owing to the steric hindrance of ATP. 

Moreover, the pore volume of nFe/Ni increased from 0.0920 cm^3^/g to 0.2479 cm^3^/g of ATP-nFe/Ni owing to the loading of nFe/Ni onto ATP (shown in Table 1), which was conducive to the rapid diffusion of p-CNB. Meanwhile, the reaction sites of nFe/Ni can be greatly increased due to the good dispersion of nFe/Ni nanoparticles. Therefore, both rates of nitroreduction and dechlorination of p-CNB can be significantly improved.

In addition, ATP, whose V_meso_ accounts for more than 90% of the V_total_, provides an anoxic environment for nFe/Ni that fills in the mesoporous of ATP, making nFe not easy to be oxidized or even passivated. This assumption is confirmed by the determination of the effective iron percentage (Fe/Fe_T_) in nFe/Ni and ATP-nFe/Ni (Table 2). It can be observed that although the percentage of Fe in nFe/Ni is higher than that in ATP-nFe/Ni, the percentage of Fe/Fe_T_ in nFe/Ni is only 50.4%, while that in ATP-nFe/Ni reaches 98.3%. In other words, the content of reductive Fe in ATP-nFe/Ni is close to 100%, indicating that only a small amount of Fe in ATP-nFe/Ni has been oxidized, due to the load of ATP. 

### 3.5. The Role of Fe in Nitroreduction and Dechlorination of p-CNB by ATP-nFe/Ni

Theoretically, Fe has three possible roles in the nitroreduction and dechlorination of p-CNB, which include acting as a donor for electron transfer and offering Fe^2+^ and H_2_ as a reductant for nitroreduction and dichlorination [23,35,36]. Both Fe^2+^ and H_2_ were produced from Fe corrosion, as shown in Equation (4).

(4) $${\mathrm{Fe}+2H}_{2}O\to {\mathrm{Fe}}^{2+}{+H}_{2}{+\mathrm{OH}}^{-}$$

In order to verify which of the three roles of Fe is the true contributor to the nitroreduction (the -NO_2_ is reduced to -NH_2_) and the dechlorination (the C-Cl bond is broken) of p-CNB, several batch experiments were conducted.

#### 3.5.1. Contribution of Fe^2+^ in Nitroreduction and Dechlorination of p-CNB

**Figure 6:**
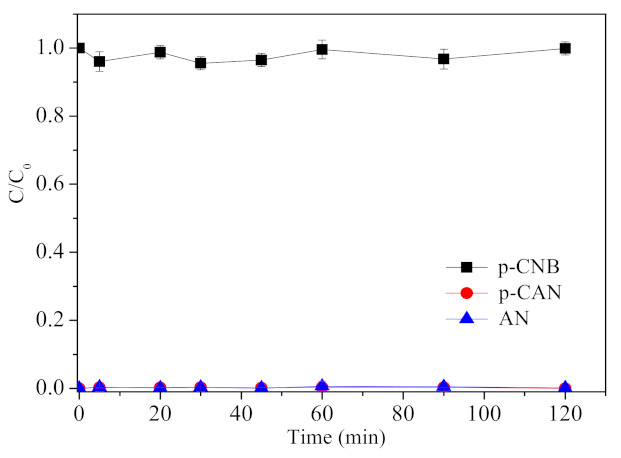
Removal, nitroreduction and dechlorination of p-CNB by FeSO_4_ (concentration of p-CNB = 10 mg/L, concentration of FeSO_4_ = 10 mmol/L and initial pH = 5.6 ± 0.1).

Figure 6 shows the removal, nitroreduction and dechlorination of p-CNB by FeSO_4_ with the concentration of 10 mmol/L. It can be observed that p-CNB could not be reduced or dechlorinated by Fe^2+^. There was a decrease of 0.1–2.1% in the concentration of p-CNB within 120 min, which could be ascribed to the adsorption of p-CNB to the iron oxide/hydroxide resulted from Fe^2+^ oxidation or precipitation [37]. 

**Figure 7:**
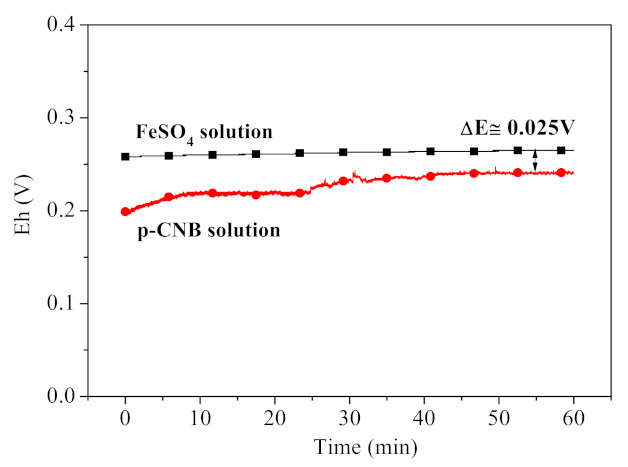
The redox potential of FeSO_4_ solution and p-CNB solution (concentration of p-CNB = 10 mg/L, concentration of FeSO_4_ = 10 mmol/L and initial pH = 5.6 ± 0.1).

The redox potential of FeSO_4_ solution and p-CNB solution were measured and presented in Figure 7. The redox potential of p-CNB solution is 0.199 V, which increases slightly with the prolonged reaction time. However, it is always below the redox potential of FeSO_4_ solution (0.258 V). Therefore, Fe^2+^ is incapable of reducing p-CNB, since it acts as an oxidant other than a reductant when it reacted with p-CNB. 

#### 3.5.2. Contribution of H_2_ in Nitroreduction and Dechlorination of p-CNB

**Figure 8:**
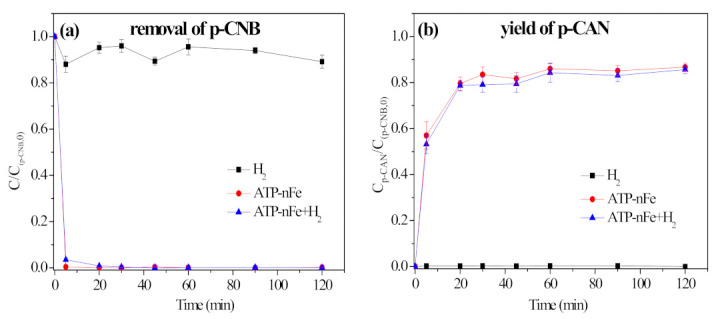

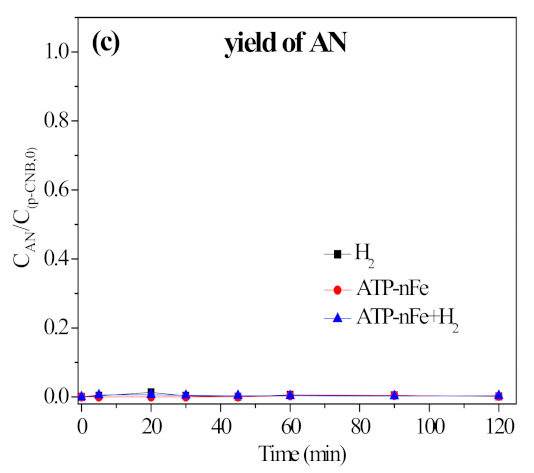
Removal of p-CNB by H_2_, ATP-nFe and ATP-nFe+H_2_ (**a**), the yield of p-CAN (**b**) and the yield of AN (**c**) (reaction conditions: concentration of p-CNB = 10 mg/L, concentration of H_2_ = 800 μg/L, dosage of material = 0.5 g/L and initial pH = 5.6 ± 0.1).

The contribution of H_2_ to nitroreduction and dechlorination of p-CNB was identified by investigating the yield of p-CAN and AN during the removal of p-CNB by H_2_, ATP-nFe and ATP-nFe+H_2_, respectively (shown in Figure 8). The addition of H_2_ was accomplished by pumping H_2_ into p-CNB solution for 30–60 min until the measured concentration of H_2_ reached 800 ug/L. It can be observed that no p-CAN or AN is formed when H_2_ is used as a reductant, but the addition of H_2_ decreases the concentration of p-CNB measured in some samples, which may be caused by the adsorption of trace p-CNB on the bubbles of H_2_ dissolved in water. When ATP-nFe reacted with p-CNB, the concentration of p-CNB dropped to nearly 0 at 30 min, and the yield of p-CAN reached 86.7% at 120 min. However, the dechlorination product, AN, is not detected. When H_2_ was bubbled into the p-CNB + ATP-nFe system, the removal of p-CNB and the yield of p-CAN were similar to that without H_2_, further suggesting that H_2_ is incapable of reducing the -NO_2_ to -NH_2_, and also could not dechlorinate p-CAN to AN, even though H_2_ can act as a reductant, theoretically.

#### 3.5.3. Contribution of Electron Transfer in Nitroreduction and Dechlorination of p-CNB 

**Figure 9:**
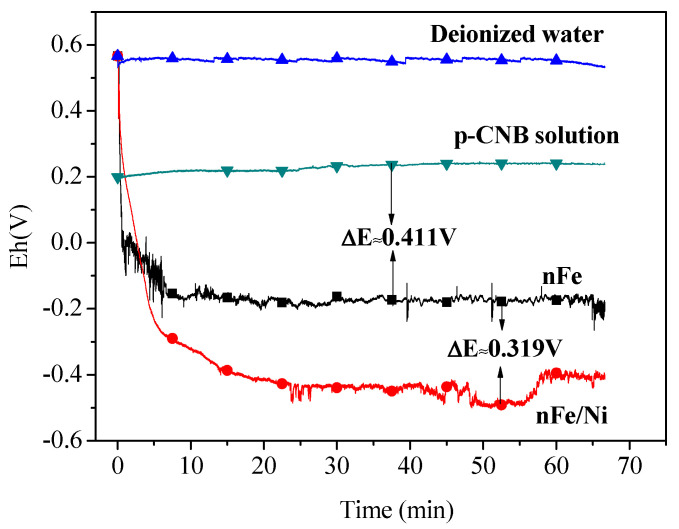
Redox potential of nFe, nFe/Ni and p-CNB solution (concentration of p-CNB = 10 mg/L, dosage of nFe (or nFe/Ni) = 0.5 g/L and initial pH = 5.6 ± 0.1).

Many studies have reported that chlorinated organic compounds can be reduced by getting electrons on the surface of nFe [38,39,40]. To investigate whether there is an electron transfer during nitroreduction and dechlorination of p-CNB, the redox potential of nFe in deionized water and p-CNB solution were measured and are shown as Figure 9. 

As can be observed from Figure 9, the redox potential of nFe decreases sharply from + 0.614 V to −0.212 V in the first 5 min of mixing with deionized water and is 0.411 V lower than that of p-CNB solution, indicating that Fe can serve as an electron donor in the nitroreduction and dechlorination of p-CNB and the electrons may be transferred from nFe to p-CNB.

### 3.6. The Role of Ni in Nitroreduction and Dechlorination of p-CNB by ATP-nFe/Ni

It can be observed from Figure 4 that when ATP-nFe/Ni reacted with p-CNB, the yield of AN was higher than those obtained by ATP-nFe. Therefore, we conducted the following experiments to reveal the roles of Ni in nitroreduction and dechlorination of p-CNB.

#### 3.6.1. Depositing on the Surface of Fe Nanoparticles to Alleviate the Oxidization of Fe 

**Figure 10:**
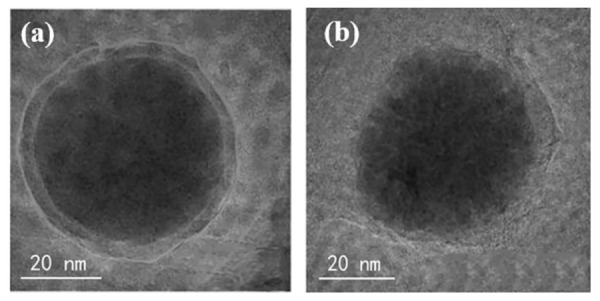
TEM images of nFe (**a**) and nFe/Ni (**b**) particles.

The TEM images of the individual Fe nanoparticle and Fe/Ni nanoparticle are shown in Figure 10. The Fe nanoparticle exhibits a core-shell structure, while an incomplete iron oxide shell is found on the surface of Fe/Ni nanoparticle. This contrasting difference can be ascribed to the fact that the surface of Fe/Ni nanoparticle is partially covered by Ni, which inhibits the direct contact of nFe with oxygen and thus prevents the oxidation of nFe to some extent. 

#### 3.6.2. Acting as the Anode of Fe-Ni Galvanic Cell to Accelerate the Corrosion of Fe 

The redox potential of nFe/Ni in deionized water is around −0.483 V (shown in Figure 9), 0.319 V lower than that of nFe, indicating that the reducing ability of nFe/Ni is stronger than that of nFe. The result can be interpreted as the formation of the Fe-Ni galvanic cell between Ni and Fe, leading to a stronger tendency of Fe to lose electrons [41,42]. The Ni plays the role of the cathode in Fe-Ni galvanic cell, which promotes the electron transfer from Fe to p-CNB. The slight increase in the redox potential of nFe/Ni after 55 min may be due to partial oxidation of Fe by dissolved oxygen. 

#### 3.6.3. Serving as a Hydrogenation Catalyst to Catalyze the Dissociation of H_2_ to H*

**Figure 11:**
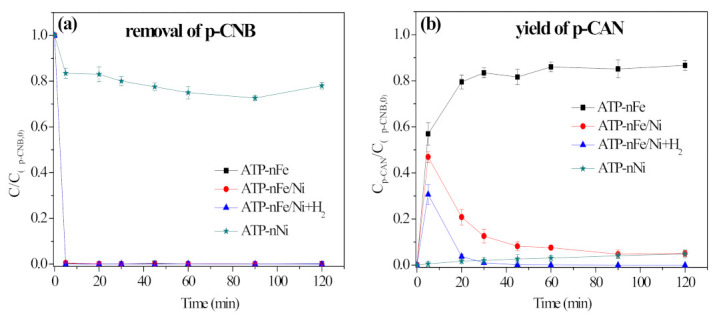

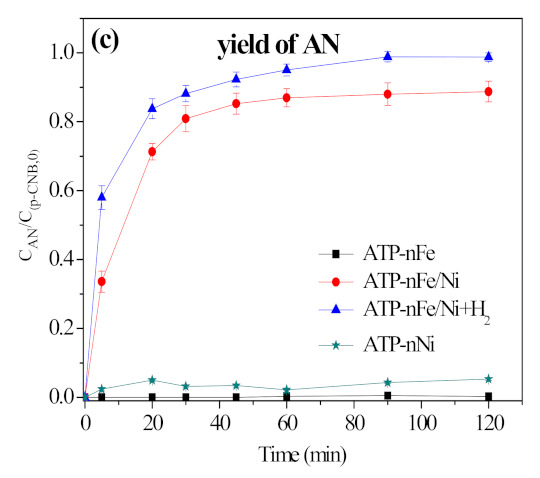
Removal of p-CNB by different materials (**a**), the yield of p-CAN (**b**) and the yield of AN (**c**) (reaction conditions: concentration of p-CNB = 10 mg/L, concentration of H_2_ = 800 μg/L, dosage of material = 0.5 g/L and initial pH = 5.6 ± 0.1).

The removal of p-CNB by different materials was conducted to examine the catalytic effect of Ni, and the results are shown in Figure 11. The removal rate of p-CNB by ATP-nNi was only 21.9% at 120 min (Figure 11a), and the yield of p-CAN and AN was 4.8% and 5.3%, respectively (Figure 11b,c). The result indicates that Ni itself has a poor ability in nitroreduction and dechlorination of p-CNB. For ATP-nFe without Ni doping, it can only reduce the -NO_2_ into -NH_2_, and cannot dechlorinate p-CNB. However, ATP-nFe/Ni can achieve an 80.9% yield of AN at the reaction time of 30 min, which should be attributed to the role of Ni. Ni has the ability to catalyze the dissociation of H_2_ produced from nFe corrosion to two active hydrogen atoms (H*), and the resulting H* can not only break the N-O bond of p-CNB to form p-CAN, but also cause the cleavage of C-Cl bond to generate AN. When H_2_ and ATP-nFe/Ni were employed at the same time, the yield of AN reached 99.5% at 90 min (Figure 11c), further confirming the catalytic effect of Ni on H_2_.

The corresponding reaction equations are shown in Equations (5)–(8) [26,43].

(5) $$H_{2}\overset{\mathrm{Ni}}{\to }2H*$$

(6) $${\mathrm{ClC}}_{6}H_{4}{\mathrm{NO}}_{2}+6H*\to {\mathrm{ClC}}_{6}H_{4}{\mathrm{NH}}_{2}{+2H}_{2}O$$

(7) $${\mathrm{ClC}}_{6}H_{4}{\mathrm{NH}}_{2}{+H*+e}^{-}\to C_{6}H_{5}{\mathrm{NH}}_{2}{+\mathrm{Cl}}^{-}$$

(8) $${\mathrm{ClC}}_{6}H_{4}{\mathrm{NO}}_{2}{+7H*+e}^{-}\to C_{6}H_{5}{\mathrm{NH}}_{2}{+\mathrm{Cl}}^{-}{+2H}_{2}O$$

Therefore, although H_2_ produced by nFe corrosion cannot cause the nitroreduction and dechlorination of p-CNB, the dissociating product H* under the catalysis of Ni not only reduces p-CNB to p-CAN, but also dechlorinates both p-CNB and p-CAN.

In addition, as discussed in Section 3.5.1 Fe can serve as an electron donor for the nitroreduction and dechlorination of p-CNB because the redox potential of Fe is 0.411 V lower than that of p-CNB. However, it can be observed from Figure 11 that the dechlorination of p-CNB by ATP-nFe does not occur. The result may be due to the electrons lost by Fe that are gained by H^+^ in p-CNB solution, and then the H^+^ transfers to the hydrogen atom (H). The resulting H has a lower energy than the H*, and therefore can only break the N-O bond with lower bond energy (201 kJ/mol) in p-CNB to form -NH_2_ (the corresponding reactions are shown in Equations (9)–(11)), but cannot break the C-Cl bond with higher bond energy (327 kJ/mol). Nevertheless, it should be possible to dechlorinate p-CNB if the hydrogen atom formed in this way has enough energy to break the C-Cl bond. These results are consistent with the findings in the reference [23]. According to their report, nFe could not dechlorinate 2,4-dichlorophenol by transferring electrons when the temperature of the reaction system was increased to 60 °C.

(9) $${\mathrm{ClC}}_{6}H_{4}{\mathrm{NO}}_{2}{+2e}^{-}{+2H}^{+}\to {\mathrm{ClC}}_{6}H_{4}{\mathrm{NO}+H}_{2}O$$

(10) $${\mathrm{ClC}}_{6}H_{4}{\mathrm{NO}+2e}^{-}{+2H}^{+}\to {\mathrm{ClC}}_{6}H_{4}\mathrm{NHOH}$$

(11) $$\;{\mathrm{ClC}}_{6}H_{4}{\mathrm{NHOH}+2e}^{-}{+2H}^{+}\to {\mathrm{ClC}}_{6}H_{4}{\mathrm{NH}}_{2}{+H}_{2}O$$

In this study, nFe reacts with p-CNB at ambient temperature, and the dechlorination of p-CNB and p-CAN (the nitroreduction product of p-CNB) does not occur even if Fe can lose electrons and theoretically transfer to p-CNB.

## 4. Conclusions

The roles of Fe, Ni and ATP in nitroreducing and dechlorinating p-CNB were investigated in this study. It was identified that the nitroreduction of p-CNB could be achieved through electron transfer (a possible action mechanism of Fe). However, the same mechanism was unable to dechlorinate p-CNB at standard temperature. Fe^2+^ was also found to be incapable of nitroreducing and dechlorinating p-CNB due to its action as an oxidant during the reaction with p-CNB. H_2_ was incapable of nitroreducing and dechlorinating p-CNB in its original state. Nevertheless, the conversion of H_2_ into H^*^ by Ni facilitated the nitroreduction and dechlorination of the p-CNB. Ni also helped to improve nFe oxidation, as well as the electron transfer of nFe. The roles of ATP included providing an anoxic environment for the nFe/Ni, reducing nFe aggregation/agglomeration during the reaction, and increasing the pore volume and reaction sites of the nFe/Ni to improve nitroreduction and dechlorination of the p-CNB. The findings in this study are critical in understanding the action mechanisms of Fe, Ni and ATP in nitroreducing and dechlorinating chloro-organic compounds.

## Figures and Tables

**Table 1:** BET specific surface area and pore parameters of ATP, nFe/Ni and ATP-nFe/Ni.

Material	S_BET_ (m^2^/g)	*S_micro_ (m^2^/g)	*S_meso_ (m^2^/g)	V_total_ (cm^3^/g)	*V_micro_ (cm^3^/g)	*V_meso_ (cm^3^/g)	Pore Size (nm)
ATP	110.16	7.15	111.04	0.3160	0.0031	0.3156	11.47
nFe/Ni	14.15	2.88	13.39	0.0920	0.0014	0.0919	26.01
ATP-nFe/Ni	101.33	36.58	77.69	0.2479	0.0188	0.2372	9.78

* S_micro_ and S_meso_ represent the specific surface areas of micropores and mesoporous, respectively; V_micro_ and V_meso_ represent the volume of micropores and mesoporous, respectively.

**Table 2:** Percentage of Fe/Fe_T_ in nFe/Ni and ATP-nFe/Ni.

Material	*Fe_T_%	*Fe%	Fe/Fe_T_%
nFe/Ni	94.2	47.5	50.4
ATP-nFe/Ni	36.2	35.6	98.3

* Fe_T_% represents the total amount of iron contained in the material; Fe% represents the amount of zero-valent iron contained in the material.

## Data Availability

All relevant data presented in the article are stored according to institutional requirements and as such are not available online. However, all data used in this manuscript can be made available upon request to the authors.
